# Calcium Sensing Receptor Expression in Ovine Amniotic Fluid Mesenchymal Stem Cells and the Potential Role of R-568 during Osteogenic Differentiation

**DOI:** 10.1371/journal.pone.0073816

**Published:** 2013-09-09

**Authors:** Pamela Di Tomo, Caterina Pipino, Paola Lanuti, Caterina Morabito, Laura Pierdomenico, Vittorio Sirolli, Mario Bonomini, Sebastiano Miscia, Maria Addolorata Mariggiò, Marco Marchisio, Barbara Barboni, Assunta Pandolfi

**Affiliations:** 1 Department of Experimental and Clinical Sciences, University “G. d’Annunzio” Chieti-Pescara, Chieti, Italy; 2 Aging Research Center, Ce.S.I., “University G. d’Annunzio” Foundation Chieti, University “G. d’Annunzio” Chieti-Pescara, Chieti, Italy; 3 StemTeCh Group Chieti, University “G. d’Annunzio” Chieti-Pescara, Chieti, Italy; 4 Department of Medicine and Aging Science, University “G. d’Annunzio” Chieti-Pescara, Chieti, Italy; 5 Department of Neuroscience and Imaging, University “G. d’Annunzio” Chieti-Pescara, Chieti, Italy; 6 Department of Comparative Biomedical Science, University of Teramo, Teramo, Italy; Tufts University, United States of America

## Abstract

Amniotic fluid-derived stem (AFS) cells have been identified as a promising source for cell therapy applications in bone traumatic and degenerative damage. Calcium Sensing Receptor (CaSR), a G protein-coupled receptor able to bind calcium ions, plays a physiological role in regulating bone metabolism. It is expressed in different kinds of cells, as well as in some stem cells. The bone CaSR could potentially be targeted by allosteric modulators, in particular by agonists such as calcimimetic R-568, which may potentially be helpful for the treatment of bone disease. The aim of our study was first to investigate the presence of CaSR in ovine Amniotic Fluid Mesenchymal Stem Cells (oAFMSCs) and then the potential role of calcimimetics in *in vitro* osteogenesis. oAFMSCs were isolated, characterized and analyzed to examine the possible presence of CaSR by western blotting and flow cytometry analysis. Once we had demonstrated CaSR expression, we worked out that 1 µM R-568 was the optimal and effective concentration by cell viability test (MTT), cell number, Alkaline Phosphatase (ALP) and Alizarin Red S (ARS) assays. Interestingly, we observed that basal diffuse CaSR expression in oAFMSCs increased at the membrane when cells were treated with R-568 (1 µM), potentially resulting in activation of the receptor. This was associated with significantly increased cell mineralization (ALP and ARS staining) and augmented intracellular calcium and Inositol trisphosphate (IP3) levels, thus demonstrating a potential role for calcimimetics during osteogenic differentiation. Calhex-231, a CaSR allosteric inhibitor, totally reversed R-568 induced mineralization. Taken together, our results demonstrate for the first time that CaSR is expressed in oAFMSCs and that calcimimetic R-568, possibly through CaSR activation, can significantly improve the osteogenic process. Hence, our study may provide useful information on the mechanisms regulating osteogenesis in oAFMSCs, perhaps prompting the use of calcimimetics in bone regenerative medicine.

## Introduction

Amniotic fluid stem (AFS) cells, isolated during pregnancy for prenatal genetic tests, have been recognized as an efficient source of cells with therapeutic potential [1]. AFS cells are widely multipotent, express some pluripotency markers and can be differentiated within the tissues of the three germ layers [1], [2]. Their properties such as low immunogenicity, the inability to form tumors, easy accessibility, and the absence of ethical problems associated with their use, make them ideal candidates for regenerative medicine [3]–[5].

Amniotic fluid-derived mesenchymal stem cells from sheep (oAFMSCs) have recently been isolated and characterized [6]. Particularly, it was demonstrated that these cells can differentiate into osteogenic, adipogenic [7] and smooth muscle lineages [6]. Moreover, sheep are considered a good animal model as they are similar to humans in size and some physiological properties. Large animals form an optimal preclinical model on which to study various diseases, such as bone disease. In this context, oAFMSCs used in allotransplantation of injured Achilles tendon led to matrix organization and tissue regeneration [8]. Furthermore, oAFMSCs have been used in tissue renovation such as the repair of diaphragmatic tendon [9] and prenatal tracheal reconstruction [10].

Again, Shaw et al. have demonstrated that oAFMSCs may be employed for autologous *in utero* stem cell gene therapy. Ovine AFMSCs obtained from sheep were transduced with GFP lentiviral vector and reinjected into the peritoneal cavity of the fetal donor. The results obtained showed the presence of GFP positive cells in many fetal organs detected by PCR, immunostaining and cytofluorimetric analysis [7].

More recently, oAFMSCs in combination with a collagen-based scaffold were used in an experimental animal study of sinus augmentation, leading to bone regeneration *in vivo* [11].

Although several studies have demonstrated the ability of AFS cells to differentiate within the injury areas, additional studies are required to increase the efficiency of osteogenic differentiation. In this connection CaSR, a G protein-coupled receptor able to bind calcium ions, may play a potentially active role. This receptor, initially cloned from bovine parathyroid, controls parathyroid hormone (PTH) secretion from the parathyroid glands in response to extracellular calcium signals [12].

Recently CaSR was found to be expressed in several tissues, such as kidney [13], the gastrointestinal tract [14], blood vessels [15], [16], bone marrow [17], mouse embryonic stem cells [18] and osteoblast cells [19]. Additionally, CaSR exercises an important role in controlling osteoblasts as well as osteoclast recruitment, differentiation and survival via multiple intracellular signals [20], [21]. Moreover, it is known that mice with cell type–specific knockout of *CaSR* display postnatal skeletal defects [22]. Thus, agonists that bind the bone CaSR may be advantageous for the treatment of bone diseases [21], [23].

Calcimimetics, such as R-568, are thought to activate G protein-linked CaSR by allosterically increasing the affinity of the receptor for Ca^2+^ leading to efficient control of uremic hyperparathyroidism [24]. Several recent studies have suggested they also possess the ability to modulate bone cell metabolism via CaSR, being therefore, of potential interest in the treatment of bone disease [23].

Today, nothing is known regarding the expression of CaSR in the model of oAFMSCs. Hence, the first aim of this work is to investigate the possible expression of CaSR in these cells. Secondly, taking into account the role of calcimimetics in bone development, our experiments were designed to test the optimal concentration of calcimimetic R-568 and its enantiomer S-568 and then investigate their selective effect through CaSR on osteogenic *in vitro* differentiation of oAFMSCs.

## Materials and Methods

### Chemicals

Powders R-568-HCl and S-568-HCl were provided by Amgen (Amgen, Inc., Thousand Oaks, CA, USA), resuspended in Dimethyl sulfoxide (DMSO) at 10 mM concentration and stored at −20°C. Calhex−231 (Santa Cruz, sc-207394) was resuspended in Ethanol at 10 mM concentration and stored at −20°C.

### Ethics Statement

All cells and tissues were collected from slaughtered animals, and this did not require an ethic statement. The uterus were collected from the slaughterhouse of the Teramo province as material used for teaching and research activity of Veterinary University course. The collection from the slaughterhouse of biological material is normed by specific conventions subscribed between the Faculty of Veterinary Medicine of Teramo and the ASL, organ of the Ministry of Health vacated to the control and safety of slaughterhouses. In each slaughterhouse the collection of tissues is controlled by an inspector which is a Veterinary that follow the regularity of all the procedures.

### Cell Cultures

The oAFMSCs were isolated and cultured as previously described [8]. Briefly, sheep fetuses were collected at a local abattoir by removing the whole pregnant uterus and bringing it at 30°C to the laboratory. Only fetuses of 25–35 cm length (approximately 3 months’ gestation) were used. Once the uterus wall was opened, ∼100 ml of amniotic fluid (AF) was collected, centrifuged at 800 g for 20 min and the resulting pellets seeded in 250 ml flasks in growth medium containing 5 ng/ml Fibroblast Growth Factor (β-FGF) as described in Colosimo et al. [8].

An aliquot of oAFMSCs was analysed, as previously described [8] for surface and intracellular stem cell markers by flow cytometry and the remaining cells were cryopreserved until use.

Cells were cultured in medium composed of Dulbecco’s Modified Eagle Medium (DMEM, PAA) with 10% Fetal Bovine Serum (FBS, PAA), 1% Penicillin/Streptomicin and 1% L-Glutamine (PAA). The medium was changed every other day. Cells were expanded when they reached 80% confluence, using 0.05% Trypsin-EDTA. All experiments were performed on cells between passages V and VI.

### Flow Cytometry

#### Antibodies

FITC-conjugated anti-CD14 and FITC-conjugated anti-CD58 were obtained from LifeSpan Bioscences (Seattle, WA, USA); anti-CD29, anti-HLA-ABC and anti HLA-DR were obtained from VMRD (Pullman, WA, USA); FITC-conjugated anti-CD31 and FITC-conjugated anti-CD45 were purchased from AbD Serotec (Oxford, UK); anti-CD49f was purchased from Beckman Coulter (Fullerton, CA, USA); anti-CD117 and anti-SOX2 were obtained from abcam (Cambridge, UK); FITC-conjugated anti-CD166 (CD166-FITC) was obtained from Ancell (MN, USA); PE-conjugated anti-Thy-1 (CD90) was obtained from Bioss (Woburn, MA, USA); PE-conjugated anti-OCT3/4 (OCT3/4-PE) was obtained from Becton Dickinson (BD, San Jose, CA); anti-TERT was purchased from Calbiochem (Gibbstown, NJ); anti-Nanog was obtained from Chemicon International (Billerica, MA). Primary antibodies were labeled using the appropriate FITC secondary antibodies (Jackson ImmunoResearch Laboratories).

#### Cell staining for flow cytometry

Staining of surface and intracellular antigens was performed as previously reported [25]. Cells were analyzed on a FACSCalibur flow cytometer (BD), using CellQuest™ software 3.2.1.f1 (BD) [26].

#### Flow cytometry measurement

Quality control included regular check-ups with Rainbow Calibration Particles (6 peaks) and CaliBRITE beads (both from BD Biosciences). Debris was excluded from the analysis by gating on morphological parameters (lymphocyte gate); 20,000 non-debris events in the morphological gate were recorded for each sample. All antibodies were titrated under assay conditions and optimal photomultiplier (PMT) gains were established for each channel. Data were analyzed using FlowJo™ v.8.8.6 software (TreeStar, Ashland, OR). The Mean Fluorescence Intensity Ratio (MFI Ratio) was calculated dividing the MFI of positive events by the MFI of negative events [27].

#### Imaging flow cytometry

Cell staining for surface or intracellular CaSR was performed as previously described for conventional flow cytometry analysis [25]. Cells were stained in both cases by a primary antibody against CaSR (1∶100, Thermo Scientific), followed by incubation with the specific FITC-labeled secondary antibody (1∶250, Jackson ImmunoResearch). Dead cells were excluded by 7-Aminoactinomycin D (7-AAD) staining (BD Biosciences), a fluorescent chemical compound that can be kept by cells characterized by damaged membranes. Sample acquisitions were performed by ImageStream (Amnis, Seattle, WA, USA; one laser, six-color configuration). To assess nonspecific fluorescence, samples stained with the respective secondary antibody alone were used. Analyses were performed by the IDEAS software (Amnis) examining 5,000 cells for each samples. Data are indicated as a percentage of CaSR positive cells in live cells whether permeabilized or not.

### Western Blotting Analysis

CaSR protein levels and β-actin were evaluated by Western blot analysis on oAFMSCs as previously described [16]. Confluent monolayer cells were cultured in 100 mm^2^ dishes, then rinsed with ice-cold PBS and scraped on ice into lysis buffer containing 50 mM Tris-HCl, pH 7.4, NP 40 1%, 150 mM NaCl and a cocktail of protease and phosphatase inhibitors (1 µg/ml each of aprotinin, leupeptin and pepstatin, 1 mM each of PMSF, Na_3_VO_4_ and NaF). Total cell lysate was either used directly for SDS-PAGE or stored at −80°C. In denaturing and reducing conditions, 80 µg of protein was used and resolved electrophoretically on linear 8% gels.

After incubation with blocking solution (5% dry milk in TBS Tween 20 0.1%) for 1h at room temperature, the blots were incubated overnight at 4°C with primary monoclonal anti-CaSR (1∶1000, Thermo Scientific) and also with a secondary goat anti-mouse IgG antibody (1∶10000, Calbiochem). The blots were finally washed and protein bands were detected using ECL Prime Western Blotting Detection Reagent (GE Healthcare). Sheep fetal calvarial derived cells (OBs) were used as positive controls.

### In vitro Treatment with Calcimimetics

Ovine AFMSCs were seeded at a density of 3000 cells/cm^2^ in 6 well plates and treated for 14 days in the presence of increasing concentrations of calcimimetics R-568 and S-568 (0.1–10 µM). In some experiments, the inhibitor Calhex-231 was pre-incubated 30 minutes before the addition of calcimimetics. To test the appropriate concentration of calcimimetics, MTT, cell number, Alkaline Phosphatase (ALP) and Alizarin Red S (ARS) assays were performed.

### MTT Assay and Cell Number

The effect of calcimimetics on oAFMSC viability was assessed by the 3-[4,5-dimethylthiazol-2-yl]-2,5-diphenyltetrazolium bromide (MTT) method. After treatment with calcimimetics as described above, a solution of MTT in phosphate buffered saline (PBS) was added to each well to a final concentration of 0.5 mg/ml. After a further 3 hours’ incubation the cells were washed and 200 µl DMSO was added for crystal solubilization. The plates were put in the dark at 37°C, and continuous gentle shaking was performed for 30 min to thoroughly dissolve the MTT dye. The spectrometric absorbance at 540 nm (for formazan dye) and 690 nm (as a background level) was read using a microplate reader (SpectraMAX 190, Molecular Devices). The effect of increased doses of calcimimetics on the number of cells was also evaluated. Cells were washed at least three times with PBS w/o Ca^++^ and Mg^++^. Then, the cells were incubated with Trypsin-EDTA at 37°C for 10 minutes and gently detached with cell scraper, centrifuged, resuspended in PBS and counted by dilution (1∶1) in Trypan blue using a Burker’s chamber. Cell counts were normalized per cm^2^.

### ALP and Alizarin Red S Assays

After treatment with different concentrations of calcimimetics (0.1–10 µM), osteogenic differentiation was assessed by ALP and Alizarin Red S staining (Sigma) at 7 and 14 days respectively using adapted published protocols [28], [29]. Briefly, for ALP activity the cells were washed twice with PBS, lysed with TRIS/HCl 10 mM pH 7.5+0.1% Triton and then incubated with a *p*-Nitrophenyl Phosphate Substrate (Sigma) for 30 min at 37°C in the dark. To stop the reaction NaOH 2N was used. ALP activity was measured by absorbance at 405 nm on a plate reader and absolute amounts of enzyme activity were calculated with p-nitrophenol (pNP, Sigma) standard. Total enzyme activity of each well was expressed as nM of pNP/mg protein/30 min.

For ARS assay, the cells were fixed in paraformaldehyde 4% for 15 minutes. Following a rinse with distilled water, the cells were stained in 40 mM Alizarin Red S (pH4.2) for 20 minutes at room temperature. For quantification of staining, 800 µL 10% (v/v) acetic acid was added to each well, and the plate was incubated at room temperature for 30 min with gentle shaking. The monolayer was then scraped from the plate with a cell scraper, transferred in 1.5 mL microcentrifuge tube and then 10% (v/v) acetic acid was added. Subsequently, 500 µL mineral oil (Sigma Aldrich) was added and samples were heated to 85°C for 10 min and then 5 min on ice. The samples were then centrifuged at 20,000 g for 15 min and 500 µL of the supernatant was transferred to a new 1.5 mL microcentrifuge tube. Then 200 µL of 10% (v/v) ammonium hydroxide was added to neutralize the acid and aliquots (150 µL) of the supernatant were read in triplicate at 405 nm in 96 well plate and normalized with respect to total cell number for each sample.

### Intracellular Calcium Measurements

Following the addition of 1 µM R- or S-568, in presence or absence of the inhibitor Calhex-231, the intracellular calcium levels in oAFMSC was measured using Fluo-4/AM (Molecular Probes, Life technologies Italia, Monza, Italy), and an inverted Olympus microscope connected to a high-speed wavelength switcher (Polychrome II; Till Photonics, Germany) and a cooled charge coupled device (CCD) camera (C6790 model; Hamamatsu Photonics, Hamamatsu, Japan). The cells were loaded with 5 µM Fluo-4/AM for 40 min at 37°C in normal external solution (NES: 140 mM NaCl, 2.8 mM KCl, 2 mM CaCl_2_, 2 mM MgCl_2_, 10 mM glucose, 10 mM Hepes, pH 7.3) supplemented with 1% (w/v) bovine serum albumin. Fluo-4/AM loaded cells were excited at 488 nm; emitted fluorescence was collected at 522 nm (one image per second). Ca^2+^ levels were reported as traces calculated as f/f_0_, where f is the fluorescence emission of a single Fluo-4-loaded cell at times ranging from 0.0 to 8 min, and f_0_ is the mean fluorescence intensity value of the same cell calculated during the first minute [30].

### IP3 Immunoassay

In the same experimental conditions described above, we quantified R-568 stimulated Inositol trisphosphate (IP3) levels in oAFMSCs using Sheep IP3 ELISA kit (BlueGene Biotech, Shangai, China). Briefly, the cells lysed (500,000 cells) were incubated for one hour together with IP3-HRP conjugate in a monoclonal anti-IP3 antibody pre-coated plate. Then, after five washing, the wells were incubated with a substrate for HRP enzyme. Finally, a stop solution was added to stop the reaction and the intensity of the color was measured spectrophotometrically at 450 nm in a microplate reader. The IP3 concentration in each sample was calculated from the standard curve.

### Statistical Analysis of Data

Data are reported as means ±SD. Statistical comparisons were made using Student’s t test. An analysis of variance was used when multiple comparisons were performed. A difference was considered significant at p<0.05.

## Results

### Ovine AFMSC Isolation and Characterization

The oAFMSCs were harvested from the amniotic cavity of sheep at mid gestation. After 5–6 days of culture the cells adhered to the plastic dish and, confirming our previous observation [6], two distinct cell populations, differing in size and shape, were recorded: small cells showing a fibroblast-like morphology and large polyhedral cells, resembling epithelial cells (data not shown). During *in vitro* expansion the fibroblast-like cells became prevalent and after the third passage of culturing the cell population appeared morphologically homogeneous with an average doubling time comprised between 12–18 hours which was not affected by cryopreservation.

Thereafter, oAFMSCs were characterized by Flow Cytometry analysis (Table 1). Confirming our previous data [8], the results indicated that oAFMSCs did not display any hematopoietic molecular markers (CD14, 31 and 45), while they expressed several surface adhesion molecules (CD29, CD58, CD166 and CD90) and intracellular stemness markers (TERT, SOX2 and Nanog). The OCT4 expression was lower than other stemness markers, while CD117 proved to be unexpressed. The absence of HLA-DR and presence of HLA-ABC suggested that these cells may be applicable in clinical transplantation settings under auto/allogenic conditions.

**Table 1:** Flow cytometry analysis of the oAFMSCs phenotype.

Antigens	Expression levels
CD117	−
CD14	−
CD31	−
CD45	−
CD49f	−
HLA-DR	−
HLA-ABC	++
CD29	++
CD58	++
CD90	++
CD166	+
TERT	+++
Nanog	+++
Oct3/4	+
Sox 2	++

– negative expression;

+/− low expression;+moderate expression;

++positive;

+++high expression.

### Ovine AFMSCs Express CaSR

**Figure 1:**
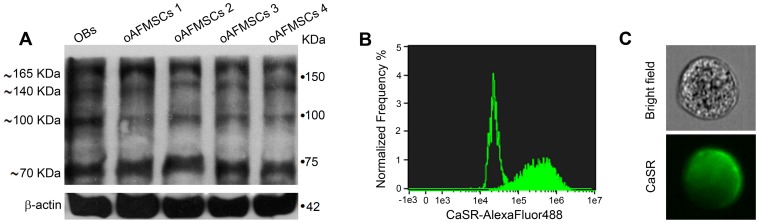
CaSR identification in oAFMSCs. (**A**) Western blot analysis shows expression of CaSR in oAFMSCs comparable to sheep fetal calvarial osteoblast (OBs) used as a positive control. β-actin was used as an internal control. Figure (**B**) shows the shift of mean fluorescence intensity of CaSR in oAFMSCs (filled histogram) as compared to the mean fluorescence intensity of secondary antibody alone (open histogram). (**C**) A representative cell in a bright field and the diffuse expression of CaSR obtained by IDEAS software (Amnis). Magnification 40x.

Ovine AFMSCs were studied for CaSR expression by western blotting and cytometry analysis. As shown in figure 1A, western blot analysis performed on four different oAFMSC samples under reducing and denaturing conditions demonstrated a pattern of bands (∼70, ∼100, ∼140 and ∼165 kDa) comparable to what was obtained in OBs, used as a positive control. These data are consistent with the results obtained from other studies reporting the expression of CaSR in various tissues [16], [19], [31].

Imaging flow cytometry in permeabilized conditions confirmed the presence of CaSR in oAFMSCs (Fig. 1B), as is also shown in a representative image (Fig. 1C) where widespread intracellular distribution of the receptor is evident.

### Effect of Calcimimetics on oAFMSCs

**Figure 2:**
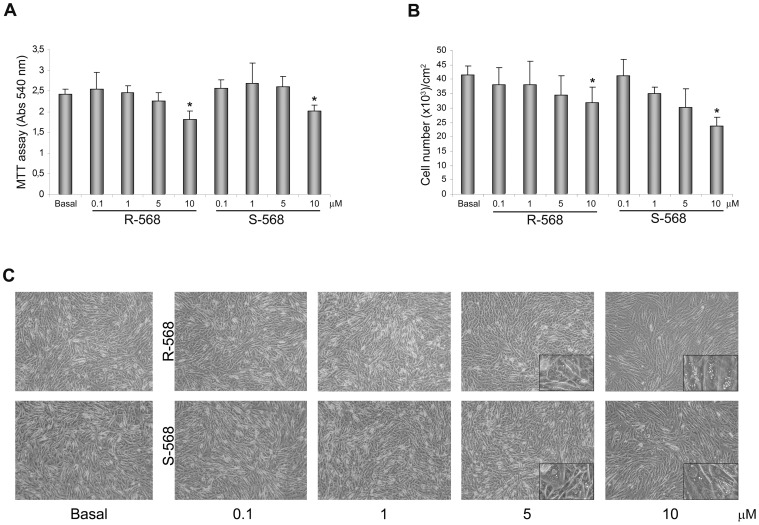
Evaluation of the optimum concentration of calcimimetics on oAFMSCs treated for 14 days. (**A**) MTT assay performed at day 14 on oAFMSCs shows a reduction in cell viability upon increasing the concentrations of R-568 or S-568 (0.1–10 µM) (*p<0.01 vs Basal). (**B**) Decreasing cell number trend on day 14 at enhanced concentrations of calcimimetics R-568 and S-568 (0.1–10 µM) (*p<0.001 vs Basal). The number of cells is normalized per cm^2^. All histograms represent the mean ± SD of three independent experiments. (**C**) Bright field images of oAFMSCs at increased concentrations of calcimimetics showing the cell morphology and the accumulations of vacuoles at high concentrations (enlargement in the right corner). Magnification 10x.

To evaluate the optimal concentration of calcimimetics, oAFMSCs were cultured in the presence or absence of increasing concentrations (0.1–10 µM) of R-568 and its enantiomer S-568. After 14 days of treatment, an MTT assay (Fig. 2A) and cell count (Fig. 2B) were performed. As evident from the histograms, calcimimetic concentrations of 0.1 and 1 µM were comparable to the basal condition and therefore not toxic to cells, while 5 µM induced a tendency to reduce cell viability, reaching statistical significance at 10 µM.

In addition, microscope analysis (Fig. 2C) confirmed the results obtained by MTT and cell counting assays. As shown by the magnification in the right-hand corners of figure 2C, we observed changes in cell morphology exclusively using 5 and 10 µM R- or S-568: oAFMSCs appeared increased in size and changed in morphology in that they were full of vacuoles. Since in these experimental conditions we also performed Oil-red O staining, this allows us to affirm that calcimimetics do not induce adipogenesis (data not shown). Thus, at high doses, these molecules can induce effective cytotoxic effects, as proven by the presence of vacuoles. Moreover, as expected, the oAFMSCs were over confluent because they had been cultured for 14 days.

### Osteogenic Effect of Calcimimetics on oAFMSCs

**Figure 3:**
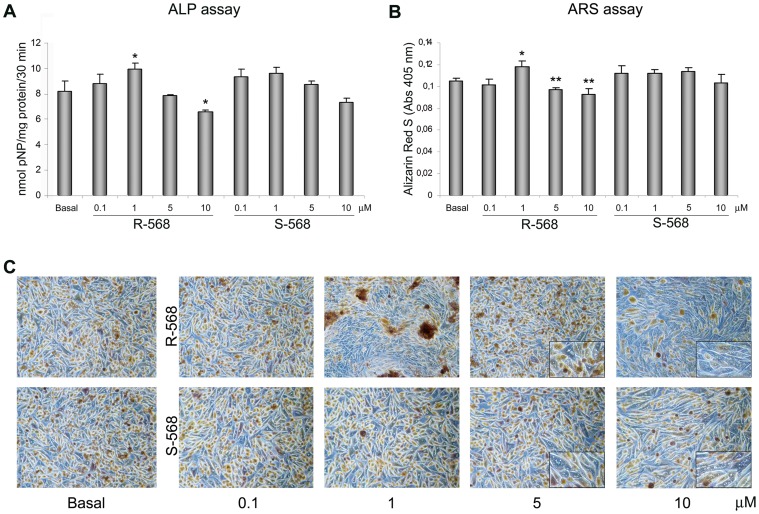
Estimation of osteogenic differentiation by Alkaline Phosphatase (ALP) and Alizarin Red S (ARS) staining. (**A**) ALP activity performed on day 7 of oAFMSC culture at increased concentrations of R-568 and S-568 (0.1–10 µM) (*p<0.05 vs Basal; **p<0.01 vs Basal). ALP activities were calculated with p-nitrophenol standard and normalized per mg of proteins. (**B**) ARS staining performed on day 14 of oAFMSC culture at increased concentrations of R-568 and S-568 (0.1–10 µM) (*p<0.01 vs Basal; **p<0.01 vs Basal). All histograms represent the mean ± SD of three independent experiments. The results are normalized per total cell number/well. (**C**) Representative images of ARS assay after 14 days of treatment with calcimimetics. Note the presence of mineral deposits after treatment with R-568 (1 µM). High concentrations (5 and 10 µM) of calcimimetics lead to a change in cell morphology with the presence of vacuoles (enlargement in the right corner). Magnification 10x.

To evaluate the potential role of R- and S-568 in osteogenesis, we performed ALP and ARS assays to assess the mineralization at different concentrations of calcimimetics (Fig. 3). As demonstrated by both ALP (Fig. 3A) and ARS assays (Fig. 3B,C), 1 µM R-568 led to a significant increase in mineral deposits (p<0.05 and p<0.01, respectively), while the enantiomer S-568 did not lead to any mineralization increase versus the basal condition. Again, we observed that both the concentrations of 5 µM and 10 µM were unhealthy to cells. All these data suggest that 1 µM R-568 may direct oAFMSCs toward osteogenic differentiation.

**Figure 4:**
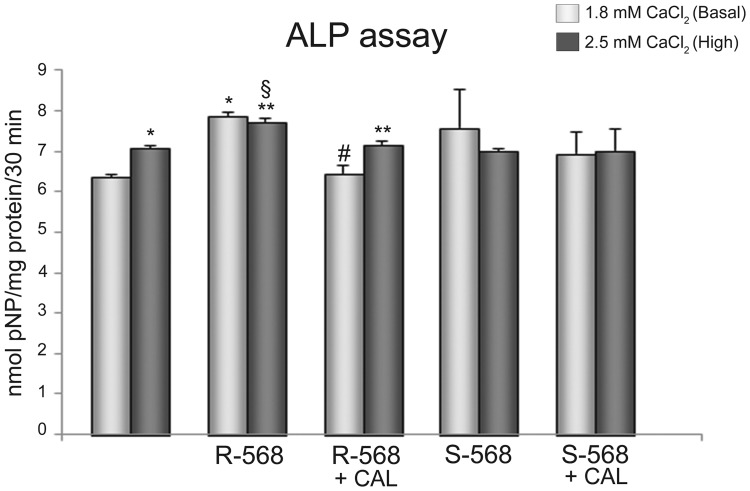
Effect of calcimimetics on ALP activity. ALP activity, detected at day 7, on oAFMSCs treated with calcimimetics pre-incubated (30 minutes) or not with Calhex-231 (1 µM, *p<0.0001 2.5 mM CaCl_2_ vs Basal 1.8 mM CaCl_2_ and R-568 vs Basal 1.8 mM CaCl_2_; ^§^p<0.01 R-568+2.5 mM CaCl_2_ vs S-568+2.5 mM CaCl_2_; **p<0.001 R-568+2.5 mM CaCl_2_ vs 2.5 mM CaCl_2_ and R-568+Calhex+2.5 mM CaCl_2_ vs R-568+2.5 mM CaCl_2_; ^#^p<0.002 R-568+Calhex vs R-568+1.8 mM CaCl_2_). ALP activities were calculated with p-nitrophenol standard and normalized per mg of proteins.

To better assess the potential calcimimetic CaSR-mediated pathway in osteogenic differentiation, we showed in Figure 4 that 1 µM R-568 increased ALP activity significantly greater than both 1.8 or 2.5 mM calcium, which is a principal agonist of CaSR receptor and thus considered a current gold standard in osteogenic process. Notably, R-568 induced ALP activity was significantly reduced by Calhex-231, a CaSR allosteric inhibitor. On the other hand, the stimulation of oAFMSCs with enantiomer S-568 (in presence or absence of Calhex-231) did not lead to any increase in ALP activity.

**Figure 5:**
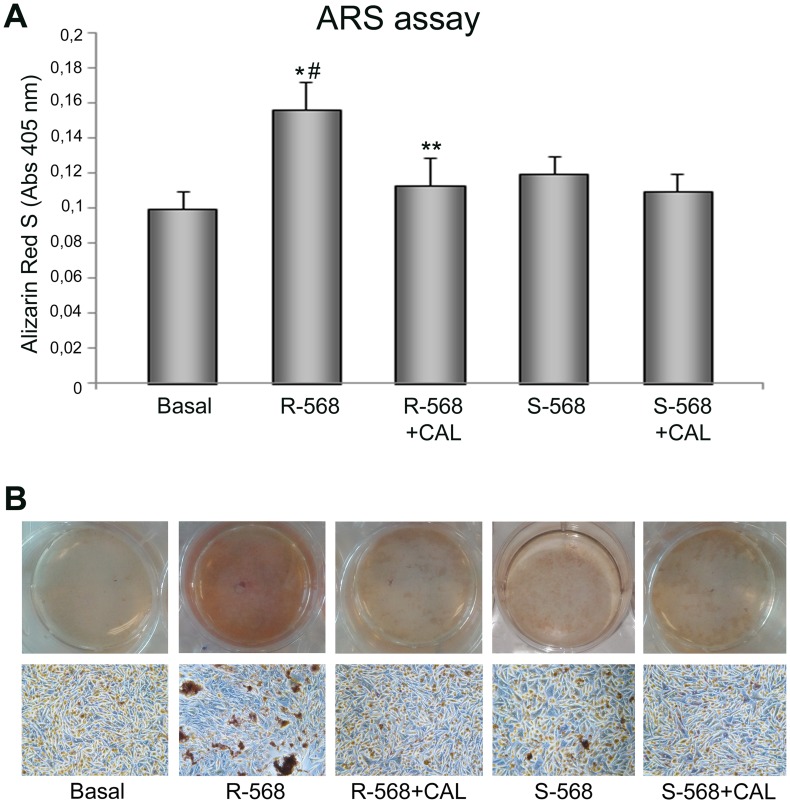
Effect of calcimimetics on ARS assay. (**A**) Ovine AFMSCs were treated with calcimimetics (1 µM) for 14 days and the mineralization was quantified at the end of treatment by reading the absorbance. The cells showed higher mineralization in response to CaSR activator R−568 (1 µM, *p<0.01), while S−568 did not lead to any mineralization. The mineral deposits produced by the culture with R−568 were significantly reduced in the presence of Calhex−231 (**p<0.05) and R-568 significantly increased ARS compared to S−568 (^#^p<0.05 R-568 vs S-568). The results are normalized per total cell number/well. (**B**) Representative full plate and microscope images of ARS assay after 14 days of treatment. Magnification 10x.

To further demonstrate the role of CaSR in R-568-induced osteogenesis in oAFMSCs, we evaluated the mineralization levels in cells treated with the CaSR inhibitor, Calhex−231 (Fig. 5A, B). As expected, the assay showed a significant R−568 increased ARS level, which significantly decreased when the cells were pre-treated with Calhex−231. Again, we did not observe any significant effects by the inhibitor on oAFMSCs treated with S-568.

Taken together these data demonstrate that the calcimimetic differentiation capabilities may be actually mediated by CaSR-pathway activation.

### Effect of Calcimimetics on CaSR Expression

**Figure 6:**
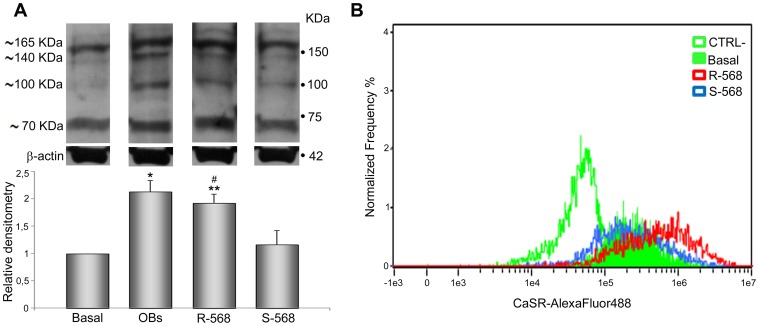
CaSR protein expression in oAFMSCs treated with calcimimetics. (**A**) Western blot analysis of oAFMSCs cultured with R-568 or S-568. Whole cell lysates were separated by SDS-PAGE, immunoblotted, and revealed with anti-CaR antibody. Equivalent amounts (40 µg) of total protein were loaded in each lane. β-actin was used as an internal control. *p<0.001 OBs vs Basal; **p<0.001 R-568 vs Basal; ^#^p<0.01 R−568 vs S−568. (**B**) Representative graph derived from Imaging flow cytometry (Amnis) shows the shift of mean fluorescence intensity (MFI) of CaSR in oAFMSCs treated with R−568 (red curve) versus basal condition (green filled curve), while treatment with S-568 did not cause any shift (blue curve). The MFI of secondary antibody alone (Alexa-488, CTRL-) is indicated by the empty green curve.

To evaluate whether the osteogenesis induced by calcimimetics is associated with increased CaSR protein levels, we performed western blotting analysis (Fig. 6A). Interestingly, as compared to basal level, we observed that in our cell model calcimimetic R-568 significantly augmented CaSR protein expression (p<0.05), which was comparable with CaSR protein levels in OBs (positive control). On the contrary, enantiomer S-568 did not show any significant effect. These data were confirmed by Imaging flow cytometry (Fig. 6B), where there is an evident shift in the mean fluorescence intensity (MFI) of CaSR in oAFMSCs treated with R−568 (red curve) compared to basal condition (green filled curve), while treatment with S-568 did not cause any such shift (blue curve).

**Figure 7:**
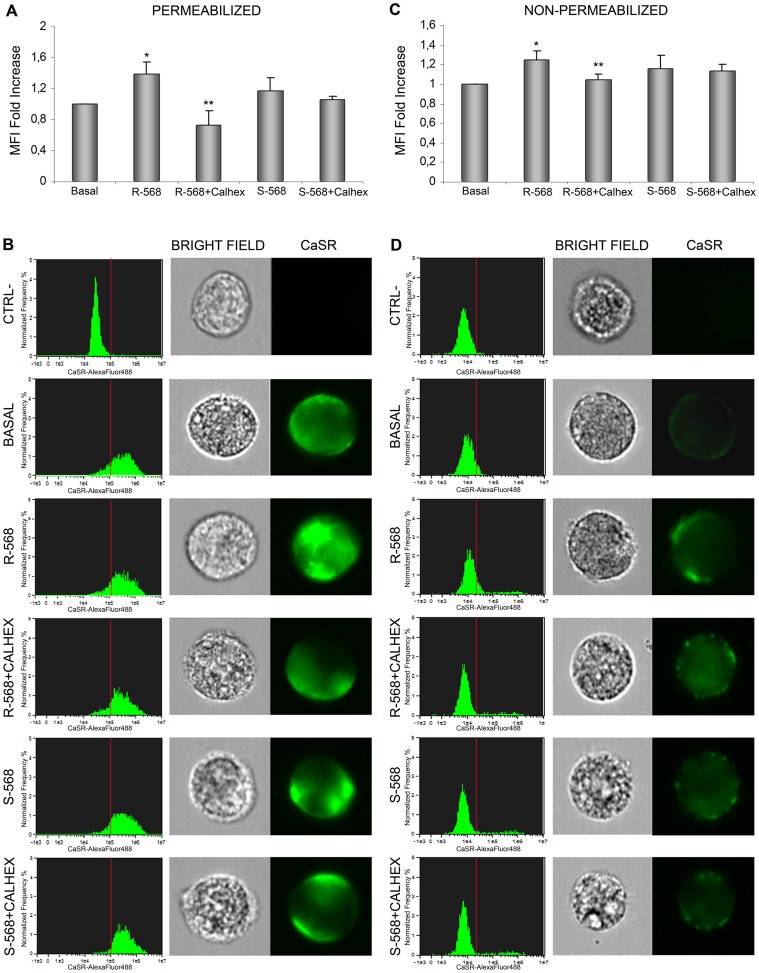
Imaging flow cytometry analysis of CaSR expression in oAFMSCs evaluated intracellularly and at membrane level. Shown here in a permeabilizing condition (**A–B**) is the CaSR expression after treatment for 14 days with R-568 or S-568 in the presence or absence of Calhex-231 (*p<0.05 R-568 vs Basal; **p<0.02 R-568+Calhex vs R-568). The histograms in **B (left),** related to the MFI bars in (**A**), show the CaSR distribution in 5×10^3^-acquired-oAFMSCs/sample. Representative single cell images in **B (right).** The same experiments were performed in non-permeabilizing conditions to detect CaSR membrane localization (*p<0.01 R-568 vs Basal; **p<0.05 R-568+Calhex vs R-568) (**C–D**)**.** Dead cells were excluded by 7-Aminoactinomycin D (7-AAD) staining. Data analyses were performed by IDEAS software (Amnis). The results shown by the bars (**A–C**) are expressed as a fold increase and are calculated by subtracting the MFI value of negative events (MFI of secondary antibody alone, CTRL-) from that of positive events. Data represent three separate experiments.

As we observed that R-568 was able to increase both CaSR protein levels and osteogenic differentiation of oAFMSCs, we investigated its potential role in the modification of CaSR cellular localization in the presence or absence of CaSR inhibitor, Calhex−231. As shown in figure 7, cells were treated in permeabilizing or non-permeabilizing conditions. In both experimental protocols we again confirmed that R−568 significantly increased CaSR levels (p<0.05), and this was reduced by Calhex−231. Treatment with enantiomer S−568, in the presence or absence of Calhex-231, did not lead to any effect on the CaSR protein expression level.

In particular, as confirmed in figure 7A, incubation with R-568 significantly increased CaSR intracellular levels (p<0.05) in a permeabilizing condition. Moreover, data shown in histograms (Fig. 7B, left) confirmed that incubation with R-568 significantly increased CaSR intracellular levels (p<0.05). Following R-568 incubation in a non-permeabilizing condition (Fig. 7C, D), we observed increased cell membrane localization of CaSR, which was significantly reversed by pre-incubation with Calhex-231. In this case too, treatment with enantiomer S-568 (in the presence or absence of Calhex-231) failed to induce any effect.

All these data suggest that calcimimetic R-568 may promote osteogenic differentiation of oAFMSCs, increasing the inner cell localization of CaSR and notably its membrane exposure, which may indicate that it has some functional role in the osteogenic process.

### Effect of Calcimimetics on Intracellular Calcium Variations and IP3 Levels

**Figure 8:**
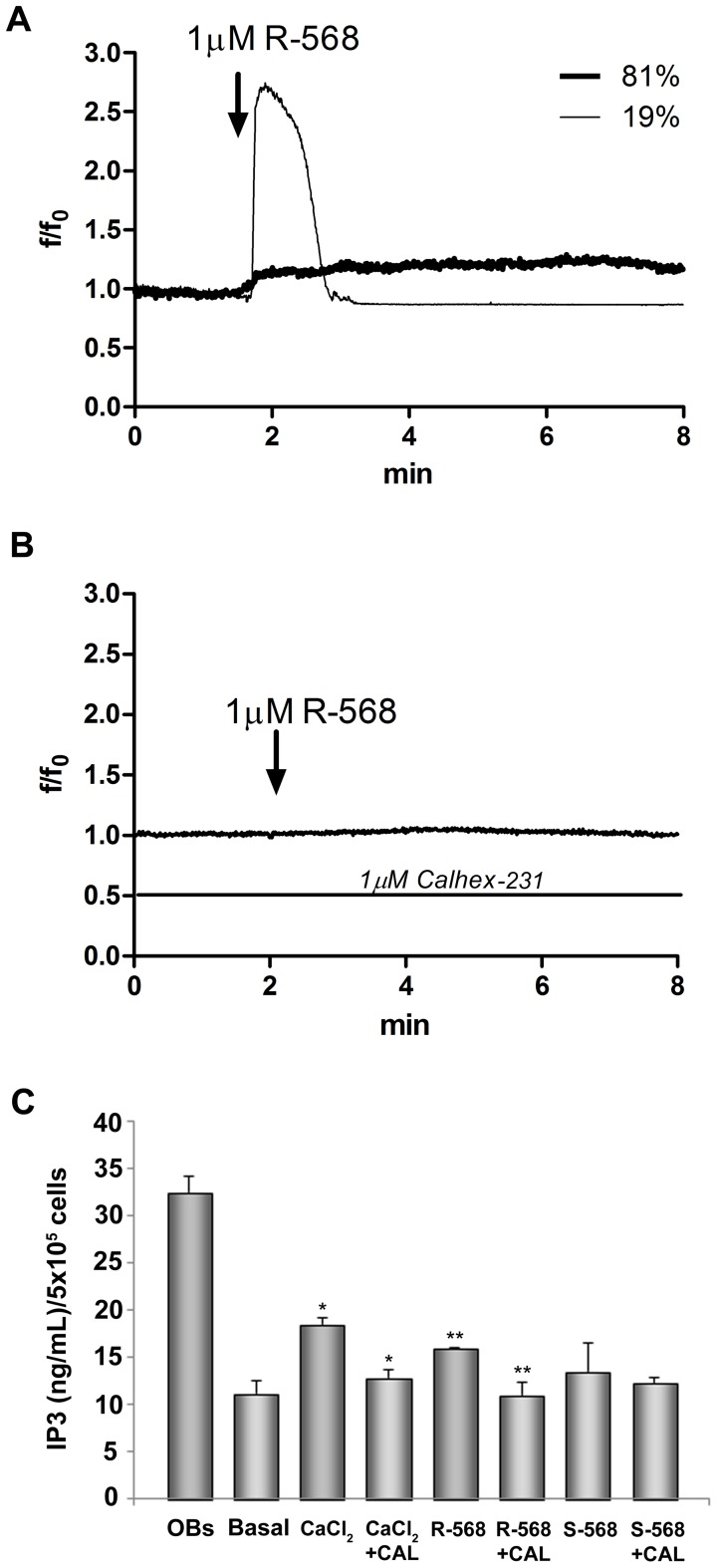
Intracellular Ca^2+^ and IP3 variations in oAFMSCs stimulated with R-568. Temporal changes in intracellular Ca^2+^ levels, expressed as f/f_0_ ratio (see Material and Methods), were measured in oAFMSCs. In panel (**A**) the representative traces showed calcium variations in cells treated with 1 µM R-568. In panel (**B**) the traces showed the cell response in presence of 1 µM Calhex-231. In panel (**C**) the IP3 levels were expressed as ng/ml/5×10^5^ cells. CaCl_2_ was used at 2.5 mM; Basal indicates 1.8 mM CaCl_2_. *p<0.02 CaCl_2_ vs Basal and CaCl_2_+Calhex-231 vs CaCl_2_; **p<0.05 R-568 vs Basal and R-568+Calhex-231 vs R-568.

To study the calcimimetic effects on CaSR functional activation, we evaluated the potential role of R-568 on intracellular calcium and IP3 level variations. As shown in Fig. 8A, R-568 (1 µM) induced an intracellular calcium increase in 19±3% of cells (28 cells out of 146 total tested cells in three indipendent fields). The cells responsive to 1 µM R-568, showed a high and transient calcium increase (19% of cells) or a low and sustained ion increase (81% of cells). When the cells were treated with 1 µM Calhex-231, no response was observed after R-568 addition (Fig. 8B). At the same concentration S-568 (1 µM, in presence or absence of Calhex-231) was not able to influence intracellular calcium levels (data not shown). Interestingly, we observed that acute stimulation (2 min) of cells with R-568 induced a significant increase of intracellular IP3 levels (p<0.05). This effect was comparable to that obtained when the cells were stimulated with the high extracellular calcium concentration, 2.5 mM. Calhex-231 (1 µM) totally abolished the R-568 effect; S-568 (1 µM, in presence or absence of Calhex-231) was not able to influence intracellular calcium levels (Fig. 8C).

## Discussion

Various different sources of mesenchymal stem cells have previously been considered attractive for orthopedic regenerative medicine, such as adipose tissue, dental pulp, or bone marrow [32], and these cells expressed a prompt osteogenic phenotype when incubated under adequate *in vitro* conditions [32], [33].

However, amniotic fluid-derived cells may have some additional biological properties that could be particularly useful toward developing a new protocol in regenerative medicine [1]. Amniotic fluid, rich in mesenchymal-like cells [34], can be easily collected during prenatal diagnosis amniocentesis without raising any ethical problems [35]. In addition AFMSCs display a high differentiation capacity toward all three germ layers demonstrated under both *in vitro* and *in vivo* conditions [1]. Due to this multi/pluripotency and to the early embryonic origin, amniotic-derived cells are considered as fetal cells, showing a degree of stemness that is typical of embryonic stem cells, even though they lack both tumorigenicity and immunogenicity [35], [36].

Our recent data demonstrated that oAFMSCs can differentiate *in vitro* into osteoblast-like cells [6], [11], as previously reported for human and rodent AFMSCs [1], [2], [37]. The fact that osteogenesis occurred was here clearly confirmed after 3 weeks’ culture in an osteogenic medium by the extensive mineralization of the extracellular matrix (data not shown).

Interestingly, in view of their therapeutic potential for bone tissue engineering, the present study shows for the first time that CaSR is expressed in oAFMSCs. It is known that CaSR is a physiological regulator of bone cell metabolism through modulation of the recruitment, differentiation and survival of osteoblasts and osteoclasts by activation of different pathways in bone cells [38].

In particular, unlike mature OB cells which are known to express CaSR [39] in totally or partially glycosylated monomers (∼140 and ∼165 kDa, considered the mature form which reaches the cell surface as a dimer of ∼300 kDa) and unlike immune-reactive polypetides of ∼70 and ∼100 kDa, we here consistently found the presence of immuno-reactive polypeptides of about 70, 100, 140 and 165 kDa in four different oAFMSC cultures (Fig. 1A).

There is substantial evidence for the implication of CaSR in the bone [20], [38] and the presence of this receptor in different bone cells including osteoblasts, osteoclasts, stromal cells, monocytes, macrophages and chondrocytes [17], [20], [40], [41]. In addition, recent works have suggested that this important receptor is expressed in stem cells such as size-sieved umbilical cord matrix equine stem cells [42], bone marrow hematopoietic stem cells [43] and mouse embryo stem cells [18].

We report here, for the first time, the presence of this functional receptor in oAFMSCs, cells known for their ability to differentiate into osteoblasts and hence be of potential use for therapeutic use *in vivo* [6], [11].

In this respect, the development of drugs able to target CaSR in bone cells may lead to an improvement in bone and cartilage regeneration. Recent studies do indeed indicate the possibility of treating bone diseases such as osteoporosis by CaSR allosteric modulators (calcimimetics and calcilytics) [21]. Interestingly, and confirming previous observations in different cellular models [39], we demonstrated by both western blot and cytometry (Fig. 6) that CaSR expression in oAFMSCs increased when the cells were treated with R-568 (1 µM). In the same experimental condition CaSR localization increased at the membrane level, potentially resulting in activation of the receptor (Fig. 7C,D). Moreover, western blot analysis (Fig. 6A) substantiated these results and gave further demonstration of what has been found up to now, namely that only R-568 treatment increased band of about 140 and 165 kDa, which may correspond to the partially and totally glycosylated monomeric form of the receptor. This represents its mature form which, when exposed on the membrane cell surface, takes on the nature of a dimer.

The calcimimetic effect on CaSR levels and cellular localization was associated with a significant increase in sheep AFMSC mineralization (ALP and ARS staining, Figs. 4 and 5), thus demonstrating a potential role for calcimimetics during osteogenic differentiation.

To confirm the potential role of CaSR in mediating the R-568 osteogenic effect we tested the effect of Calhex-231, a CaSR allosteric inhibitor; this totally reversed R-568 induced mineralization. One notes that treatment with enantiomer S-568, in the presence or absence of Calhex-231, had no effect on either mineralization or the CaSR protein expression level (Figs. 5 and 6). Moreover, we studied the R-568 effects on CaSR functional activation evaluating pathways usually activated by extracellular high calcium level. Interestingly, we observed that acute stimulation of cells with R-568 induced a significant increase of both intracellular calcium and IP3 levels (Fig. 8).

Taken all together, these results suggest that not only is CaSR more expressed in R-568 treated cells, but its augmented expression at a membrane level is associated to its activation, indicating an increased functionality: thus it may actively contribute to the process of osteogenesis.

This finding is in agreement with reports on other cells, such as the human osteoblastic MG-63 cell line [19] in which it has been observed that CaSR is not only expressed but is functionally active in osteoblast plasma membrane.

Our results strongly suggest that agonists binding bone CaSR may be advantageous for the treatment of bone injury [21], [23].

Although Barradas et al. have recently shown that calcium-enhanced osteogenic differentiation in bone marrow-derived mesenchymal stromal cells is not mediated by CaSR activation [44], our results are in accordance with part of the literature in demonstrating that an elevation of Ca^2+^_o_ concentration, as well as the use of calcimimetics, strongly activates functional CaSR in several cell lines [39], [42], [45].

One should note that calcimimetics may have a favorable effect on bone cells expressing CaSR: they are not able to potentiate the effects of extracellular calcium on osteoblasts and osteoclasts in the absence of CaSR [46] but are efficacious in cells expressing the receptor [47].

Another noteworthy point we have demonstrated is that at a low and not cytotoxic concentration of 1 µM, calcimimetic R-568 significantly increases osteogenic differentiation in control culture conditions. Different data were obtained when S-568 was examined at the same concentration, showing that there is no effect of S-568 in the osteogenic differentiation process. The effect of R-568 on osteogenesis is specific.

In conclusion, we have shown for the first time that not only is CaSR expressed in oAFMSCs, but its expression and function are significantly increased by calcimimetic R-568, which is also able to induce polarization of the receptor up to the membrane; this may indicate a helpful role by this calcimimetic in CaSR activation and hence possible implication of it in osteogenic differentiation. Further studies will be necessary to evaluate the potential basal and stimulated role of CaSR in human AFMSCs, since drugs targeting CaSR may be used in addition to boost movement of transplanted stem cells to the site of injury, entailing significant clinical implications for stem cell transplantation.
